# Transcriptional Profiling of Abomasal Mucosa from Young Calves Experimentally Infected with *Ostertagia ostertagi*

**DOI:** 10.3390/ijms26052264

**Published:** 2025-03-04

**Authors:** Clarissa Boschiero, Ethiopia Beshah, Mariam Bakshi, Eliseo Miramontes, Deborah Hebert, Peter C. Thompson, Cong-Jun Li, Xiaoping Zhu, Dante Zarlenga, George E. Liu, Wenbin Tuo

**Affiliations:** 1 Animal Parasitic Diseases Laboratory, BARC, Agricultural Research Service, USDA, Beltsville, MD 20705, USA; 2 Department of Veterinary Medicine, University of Maryland, College Park, MD 20742, USA; 3 Animal Genomics and Improvement Laboratory, BARC, Agricultural Research Service, USDA, Beltsville, MD 20705, USA

**Keywords:** cattle, gastrointestinal, abomasum, mucosa, *Ostertagia ostertagi*, immunity, nutrition, RNA-seq

## Abstract

*Ostertagia ostertagi*, also known as the brown stomach worm, causes significant pathology in the abomasum, resulting in production and nutritional losses in cattle. Alternative control measures, such as vaccination, are urgently needed because of rapidly growing anthelmintic drug resistance. There is a need to understand host responses to the infection, especially immune responses, to advance vaccine discovery and design. Therefore, the present study investigated comprehensive changes in gene transcription in the abomasal mucosa of cattle infected with *O. ostertagi* at 0, 3–5, 7–9, 10, and 21 days post-infection (dpi) using RNA sequencing (RNA-seq). Compared to uninfected controls, infected animals exhibited significant increases in differentially expressed genes (DEGs) throughout the infection period. Infection induced more upregulated than downregulated genes in the abomasal fundic mucosa (FUN) when compared to the abomasal pyloric mucosa (PYL). The largest transcriptional changes occurred between 7–9 and 10 dpi during the final development of the L4 and their emergence from the gastric glands. Most DEGs are associated with host immunity, cellular reorganization, cell migration, and proliferation. Tuft/epithelial cell response to the infection was atypical, lacking an anticipated increase in key alarmin cytokine genes. Numerous genes associated with T helper (Th) 1, Th2, and Th17 responses and T cell exhaustion were upregulated, suggesting altered immune regulation. The data collectively indicate that *O. ostertagi* infection elicits massive host responses, particularly immune responses, which are intertwined with the parasite’s disruption of abomasal function, which likely impairs the nutrient utilization of the host. The infection is characterized by the absence of a dominant Th response and displaying a mixed activation of Th1, Th2, and Th17 pathways. Elevated expression of T cell exhaustion genes and lack of increase in epithelial alarmin cytokine genes suggest a downregulation of, or a deficiency in initiating, effective host immunity to the infection. Understanding mechanisms of parasite-mediated immune evasion and their nutritional consequences will facilitate the rational design of protective vaccines against infections of complex nematode parasites.

## 1. Introduction

Endemic infections by parasitic helminths remain a major obstacle to cattle production worldwide [1]. Anthelmintic drugs were highly efficacious for decades until the recent widespread emergence of drug resistance, which prompted the need for alternative control strategies to reduce worm burdens in livestock. Vaccines would provide cost-effective control but have been difficult to develop due to the complexity of multicellular parasites. Understanding the interactions between the host and parasites at the site of infection is critical for rational vaccine design strategies.

Ostertagiasis, caused by the nematode parasite *Ostertagia ostertagi*, represents one of the most pathogenic and economically important nematode diseases of cattle in temperate regions of the world. Common clinical symptoms of ostertagiasis in calves include altered gastric functions, reduced gastrointestinal (GI) motility, elevated abomasal pH, mucosal hyperplasia, diarrhea, weight loss, and reduced feed intake and weight gain [2]. When grazing, cows ingest the stage 3 larvae (L3) of the worm, which reach the abomasal gastric glands in less than a day post-infection (dpi). Over 3–5 dpi, L3 in the gastric glands molt to become L4. The L4 continues to develop until the final molt to adult worms around 10 dpi. The adult worms then exit the gastric glands and remain in the abomasal lumen for weeks to months, mating and producing eggs, which then pass out in feces to the pasture. In the abomasum of the infected animal, developing larvae and adult worms cause severe pathology, resulting in clinical symptoms. Among prior investigations of immune responses to GI nematodes, no previous attempt has made a careful and holistic examination of the host responses in the abomasum to *O. ostertagi* infection. Previous investigations of the expression of immune molecules by quantitative polymerase chain reaction (PCR) have helped profile the upregulation of T helper (Th) responses to the infection. However, protective immunity is very slow to develop, and animals remain susceptible to the nematode parasites even after multiple grazing seasons of exposure. This suggests these parasites manipulate host immune responses and evade host immunity [3,4]. The intricate cellular responses of cattle abomasal mucosa to *O. ostertagi* infection as the nematode develops may hold the key to understanding the establishment of protective immunity.

Differential immune responses were observed in diverse parts of the abomasum in sheep when infected with the related gastrointestinal nematode, *Haemonchus contortus* [5]. Clear differences in immune cell populations defined the pyloric (PYL) and fundic (FUN) abomasal tissues based on antibody staining and indicated region-specific responses within the abomasum. Similarly, peripheral blood monocytes (PBMCs) isolated from resistant and susceptible sheep responded differently to *H. contortus* antigens [5,6], suggesting an important role for immune cells in controlling GI nematodes in the ruminant gut. Multiple researchers have reported a role for toll-like receptors in mediating host responses to *Ostertagia* species [7,8].

Transcriptional analysis by RNA-seq of host genes is one of the most powerful and holistic methods to investigate an overall differential gene expression pre and post-infection. However, few studies have used RNA-seq to explore the global gene expression of the abomasum tissues at different stages of nematode infection. One report described bovine host responses to primary and repeated infections by *O. ostertagi* using microarray technology, employing a panel of significantly regulated genes [9]. Subsequent research on gene expression in cattle infected by *O. ostertagi* was conducted using PCR but was limited to panels of genes deemed important in host resistance [3,4,10,11]. In Angus cattle, abomasal transcriptomic responses to infection by GI parasites (*O. ostertagi* and *Cooperia oncophora*) were studied in the fundic abomasa [12]. Another study in cattle showed transcriptomic responses on intestinal samples infected with *C. oncophora* [13].

Here, a time-course study that spans the entire parasitic life cycle of *O. ostertagia* within the host was conducted to investigate the global cellular responses of tissues from PYL and FUN regions of the cattle abomasum. RNA-seq was used to characterize differentially expressed genes across five time periods corresponding to different developmental stages of the infecting worms. Particular emphasis is placed on the significant immune responses found, but care is taken to outline the biological pathways most affected in these tissues and the co-expression of functional gene modules. This is the first analysis of the global host responses in the gastric mucosa to *O. ostertagi* infection, which provides an overall transcriptomic basis for the analysis of novel defense mechanisms.

## 2. Results

### 2.1. Mapping Reads

The sequence data had a maximum read length of 150 bp using a paired-end format and resulted in an average of 51,512,133 raw reads (Table S1). Once filtered, an average of 46,120,147 clean reads per sample were obtained, indicating that ~89.5% passed the filtering step (Table S1). Clean reads were aligned with the *Bos taurus* ARS-UCD1.3 reference genome [14], with an average mapping rate of 96.52% for all samples (Table S1). The 927,023,554 reads were mapped to unique genome locations where ~89% were assigned to annotated regions of the cattle genome, and the remaining ~11% were not assigned to any annotated genome locations or assigned to multiple genomic locations (Table S1). The reads that uniquely mapped to the cattle reference genome ranged from 85.66–91.79% (Table S1), while ~7.15% were aligned to multiple locations. Reads mapping to unique genome locations and assigned to annotated regions of the cattle genome were used for downstream bioinformatic analysis.

### 2.2. Quality Assessment of the Samples

**Figure 1:**
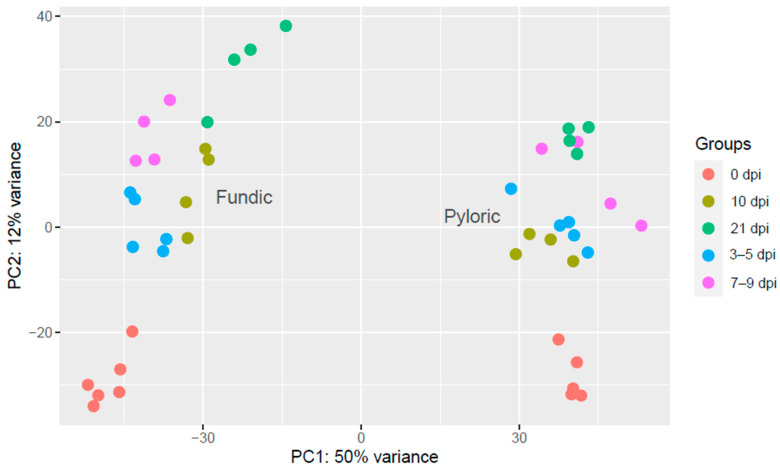
Principal component analysis (PCA) plot of 45 cattle samples with five different time points and two tissue types (fundic (FUN) and pyloric (PYL) mucosa). The PCA plot was based on the normalized counts using DESeq2. The PCA plot shows the sample-to-sample distance for normalized gene counts at five time points (0, 3–5, 7–9, 10, and 21 dpi). Each time point has a different color.

PCA was employed to assess read quality using normalized counts, knowing that well-controlled studies typically cluster replicate samples closely. Our PCA plot revealed distinct clusters separating FUN from PYL; additionally, each sampling date further subdivided each tissue type (Figure 1). Tissue type (the first principal component) explained 50% of the total variance, and sample date (the second principal component) explained 12% of the variance (Figure 1). In addition, the PCA plots showed that the replicates for each time point clustered closely for each tissue. Based on the PCA distribution patterns, 3 and 5 dpi animals were combined as the 3–5 dpi group, and 7 and 9 dpi animals were combined as the 7–9 dpi group. This provided greater replication for each of the groups.

### 2.3. Differentially Expressed Genes (DEGs)

**Figure 2:**
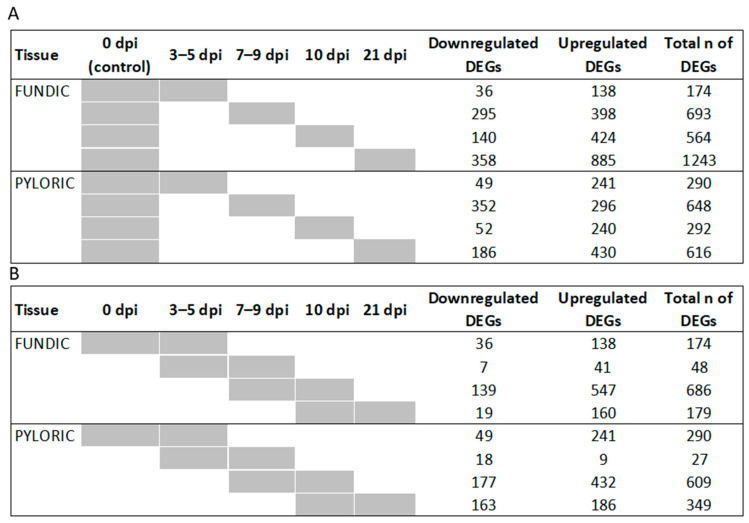
The number of differentially expressed genes (DEGs) at different time points in cattle fundic (FUN) and pyloric (PYL) mucosal tissues. Up- and downregulated genes were observed at four time points (3–5, 7–9, 10, and 21 dpi) when compared to 0 dpi (control) (**A**) and at four neighboring time junctions (0 vs. 3–5 dpi, 3–5 vs. 7–9 dpi, 7–9 vs. 10 dpi, and 10 vs. 21 dpi) (**B**).

DEGs (FDR < 0.05 and |log_2_FC| > 2) were identified using DESeq2 [15] in FUN or PYL samples at four time points (3–5, 7–9, 10, and 21 dpi) (Figure 2A, Table S2). Using 0 dpi as a baseline (control), infection induced more gene upregulation than downregulation in FUN at all four time points (3–5, 7–9, 10, and 21 dpi). The same is true for PYL, except for 7–9 dpi. Using 0 dpi as the baseline, the most DEGs were observed in FUN at 21 dpi (1243), followed by 7–9 dpi (693 DEGs), 10 dpi (564 DEGs), and 3–5 (174 DEGs). For PYL, the greatest number of DEGs occurred at 7–9 dpi (648 DEGs), followed by 21 dpi (616 DEGs), 10 dpi (292 DEGs), and 3–5 dpi (290 DEGs).

**Figure 3:**
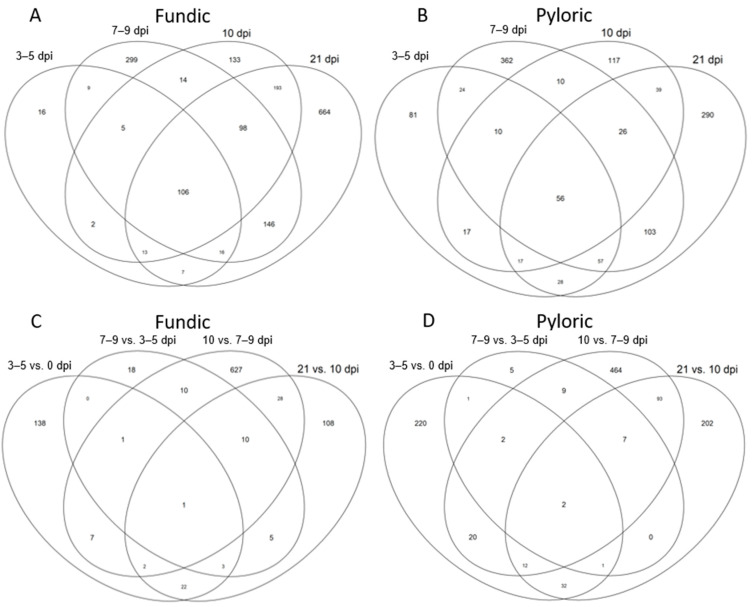
Venn diagram plots comparing DEGs at different time points in each tissue. (**A**). Four different time points, 3–5, 7–9, 10, or 21 dpi, were compared to 0 dpi (control) in cattle fundic (FUN) mucosal tissue. (**B**). Four different time points, 3–5, 7–9, 10, or 21 dpi, were compared to 0 dpi (control) in cattle pyloric (PYL) mucosal tissue. (**C**). Neighboring time points comparison, 0 vs. 3–5, 3–5 vs. 7–9, 7–9 vs. 10, and 10 vs. 21 dpi, in cattle fundic (FUN) mucosal tissue. (**D**). Neighboring time points comparisons, 0 vs. 3–5, 3–5 vs. 7–9, 7–9 vs. 10, and 10 vs. 21 dpi, in cattle pyloric (PYL) mucosal tissue.

In addition, the DEGs (FDR < 0.05 and |log_2_FC| > 2) were identified between proximal time points in both FUN and PYL (Figure 2B, Table S3). More upregulated than downregulated DEGs were characteristic of FUN at all time points. For PYL, more upregulated than downregulated DEGs were also identified for all time points, except for the 7–9 dpi vs. 3–5 dpi comparison. For both FUN and PYL in neighboring comparisons, the 10 dpi vs. 7–9 dpi had the highest number of DEGs, totaling 686 and 609 DEGs, respectively (Figure 2B). Subsequently, the DEGs were compared to identify shared and unique genes among time points in each tissue and between tissues across time points. Again, the comparisons were conducted using 0 dpi as the non-infected control or by comparing immediate neighboring time points (Figure 3A,B, Tables S4 and S5) (Figure 3C,D). Employing 0 dpi DEGs as the uninfected control (Figure 3A,B), we identified more unique DEGs in FUN at 21 dpi (664 DEGs) than at any other time points (Figure 3A); by contrast, the most unique DEGs in PYL occurred at 7–9 dpi (362 DEGs) and 21 dpi (290 DEGs) (Figure 3B).

In FUN, shared DEGs were especially abundant when comparing 7–9 dpi to 21 dpi (146 DEGs) and 10 dpi to 21 dpi (193 DEGs). In PYL, shared DEGs were most abundant when comparing 7–9 and 21 dpi (103 DEGs) (Figure 3A,B). Infection induced shared genes of 106 and 56 DEGS across all time points (when compared to uninfected calves at 0 dpi) in FUN and PYL, respectively (Figure 3A,B). Of these 106 shared fundic DEGs, 61 (57.5%) are related to immune/inflammation responses, and 22 (20.8%) are unannotated (Table S4). Of the 56 shared pyloric DEGs, 35 (66%) are related to immune/inflammation responses, and 14 (25%) are unannotated (Table S5).

Overall, FUN at 21 dpi had the most unique genes across all time points (Figure 2A), whereas PYL at 7–9 dpi had more unique genes when compared to other time points (Figure 3B).

When DEGs from the immediate neighboring time points were compared (Figure 3C,D), 7–9 dpi vs. 10 dpi (629 DEGs) had the highest number of unique DEGs, while 0 dpi vs. 3–5 dpi (138 DEGs) or 10 vs. 21 dpi (108 DEGs) also had a significant number of unique DEGs in FUN (Figure 3C). In PYL, high numbers of unique DEGs were evident at all time points except for 3–5 dpi vs. 7–9 dpi comparisons (5 DEGs) (Figure 3D). Lower numbers of shared DEGs (~30 or fewer) were observed in both FUN and PYL at all time points except for the comparison between 7–9 vs. 10 dpi and 10 vs. 21 dpi (93 DEGs) (Figure 3C,D).

**Figure 4:**
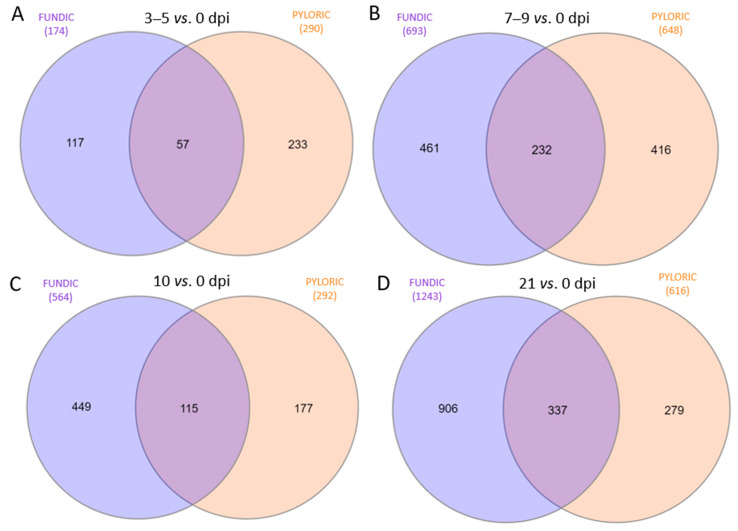
Venn diagram plots comparing DEGs between fundic (FUN) and pyloric (PYL) mucosa at four different time points: (**A**) 3–5 vs. 0 dpi; (**B**) 7–9 vs. 0 dpi, (**C**) 10 vs. 0 dpi, and (**D**) 21 vs. 0 dpi.

Shared and unique genes were also analyzed between FUN and PYL between the neighboring time points (Figure 4). While there is a general trend for the shared genes, unique genes of FUN were dramatically increased over time (Figure 4).

Volcano plots depict the most significantly expressed DEGs by each tissue across all time points (Figure S1). The top 50 upregulated DEGs in FUN at 3–5 dpi included those related to phagocytosis (*collectin-46-like* [*LOC112444733*], *CL43* and *CL46*), extracellular matrix organization and inflammatory response (*MMP1*, *MMP3*, *MMP7*, and *MMP12*); the 43 downregulated DEGs in FUN at 3–5 dpi had no clear trend of responses (Figure S1, top first panel; Table S2). The top 50 upregulated DEGs in PYL at 3–5 dpi had genes relating to granulocyte migration and host immune responses (*CXCL13*, *CCL19*, *TREM1*, *BOLA-DYA*, *C4BPA*, *ELEFIN* [*LOC100850808*], and *keratin-associated protein 10-6* [*LOC515676*]). Again, the top 50 downregulated genes in PYL at 3–5 dpi showed no clear trend of responses (Figure S1, top second panel; Table S2). The top 50 upregulated DEGs in PYL at 7–9 dpi continued to emphasize genes pertinent to phagocytosis and granulocyte migration (*collectin-46-like* [*LOC112444733*], *CL43*, *CL46*, and *TREM1*) and also immune responses (*ITLN2*, *intelectin-2* [*LOC100336682*], *beta-defensin 103B-like* [*LOC789175*], *T cell receptor beta variable 6-4-like* [*LOC101907820*]). The top 50 downregulated DEGs in PYL at 7–9 dpi also had no collective trend in responses. They include a large number of unmapped/unannotated genes (Figure S1, top third panel; Table S2). The top upregulated DEGs in PYL included genes relevant to granulocyte migration and host defense (*PF4*, *TREM1*, *GPR33*, *APOBEC3Z1*, *C4BPA*, and *relaxin-3 receptor 1* [*LOC530472*]). Many unannotated genes in the top 50 downregulated DEGs at 7–9 dpi precluded the determination of a clear functional trend. The top 50 upregulated DEGs in 10 dpi FUN almost replicated the response at 3–5 dpi, enriched for genes involved in immune and inflammatory response, and phagocytosis genes (*CL43*, *CL46*, *collectin-46-like* [*LOC112444733*], *MMP3*, *MMP7*, *MMP12*, *MMP13*). The top 50 upregulated DEGs of 10 dpi PYL showed genes related to immune responses such as *ITLN2*, *LOC508646*, *IDO1*, *LOC100336682*, *LOC100139916*, *LOC100301305*, *LOC100139670*, and *IFI44* (Table S2). In 21 dpi FUN, the top upregulated 50 DEGs contained genes consistent with inflammation, phagocytosis, and antibacterial activity (*collectin-46-like* [*LOC112444733*], *LOC100300483*, *CL43*, *CL46*, and *KNG1*), inflammatory response (*MMP3*, *MMP7*, *MMP9*, and *MMP12*), and granulocyte activity (*PRG3*). In 21 dpi PYL, the top 50 upregulated DEGs had genes important for tissue homeostasis, granulocyte migration, and host defense (*collectin-46-like* [*LOC112444733*], *CL46*, *SLC28A2*, *PF4*, *TREM1*, *APOBEC3Z1*, *granzyme B* [*LOC508646*] and *DUOX1*), and immune responses (*C4BPA*, *IGHG1*, *immunoglobulin heavy variable 4-59-like* [*LOC112443200* and *LOC112443201*], and *beta-defensin 103B-like* [*LOC789175*]).

In addition, genes related to specific categories of immune responses were searched for differential expression in FUN and PYL at different time points (Table S6). Six categories, including T cell exhaustion (TCE), Th1, Th2, Th17, interleukin, and Tuft cell-specific genes, were analyzed. Several TCE genes were identified in FUN and PYL, totaling 23 and 19 DEG genes, respectively. Most of the TCE DEG were upregulated in both mucosal tissues. Th1, Th2, and Th17 categories revealed 15 DEGs in FUN and 7 in PYL, all of which were upregulated DEGs. Interleukin genes were upregulated in both mucosal tissues, with 21 in FUN and 12 in PYL.

### 2.4. GO and KEGG Enrichment Analysis of DEGs

**Figure 5:**
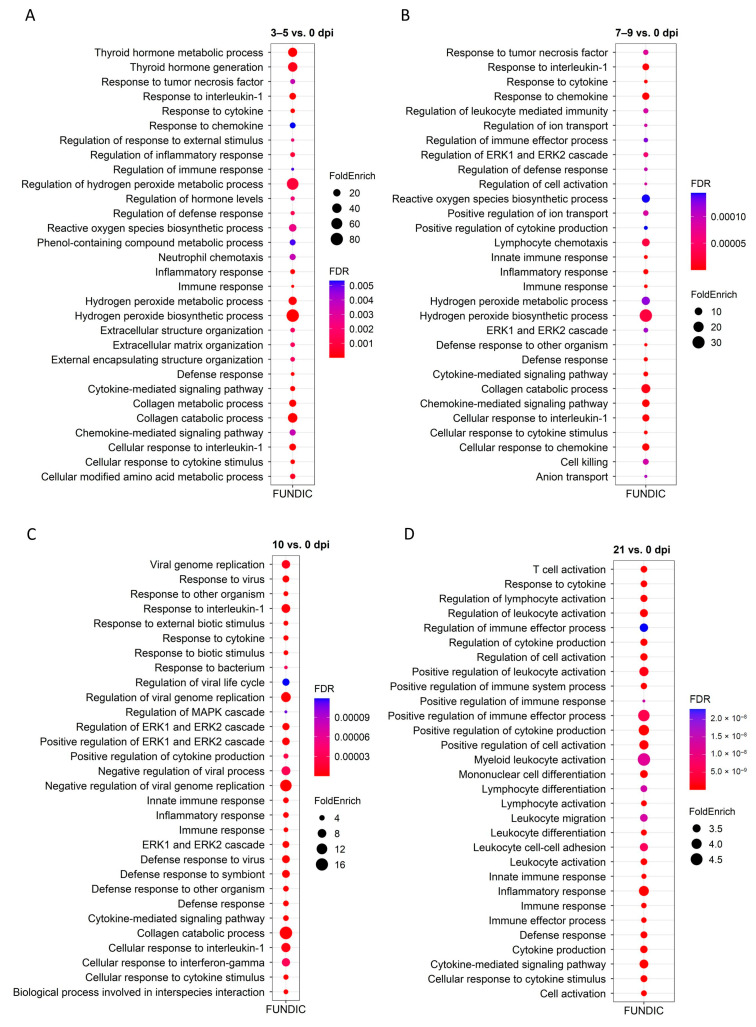
Top 30 enriched GO pathways for differentially expressed genes (DEGs) of cattle fundic (FUN) mucosal tissue at four time points (3–5, 7–9, 10, and 21 dpi) compared to 0 dpi (control). (**A**), 3-5 vs. 0 dpi; (**B**), 7–9 vs. 0 dpi; (**C**), 10 vs. 0 dpi; (**D**), 21 vs. 0 dpi. FoldEnrich: Fold Enrichment.

**Figure 6:**
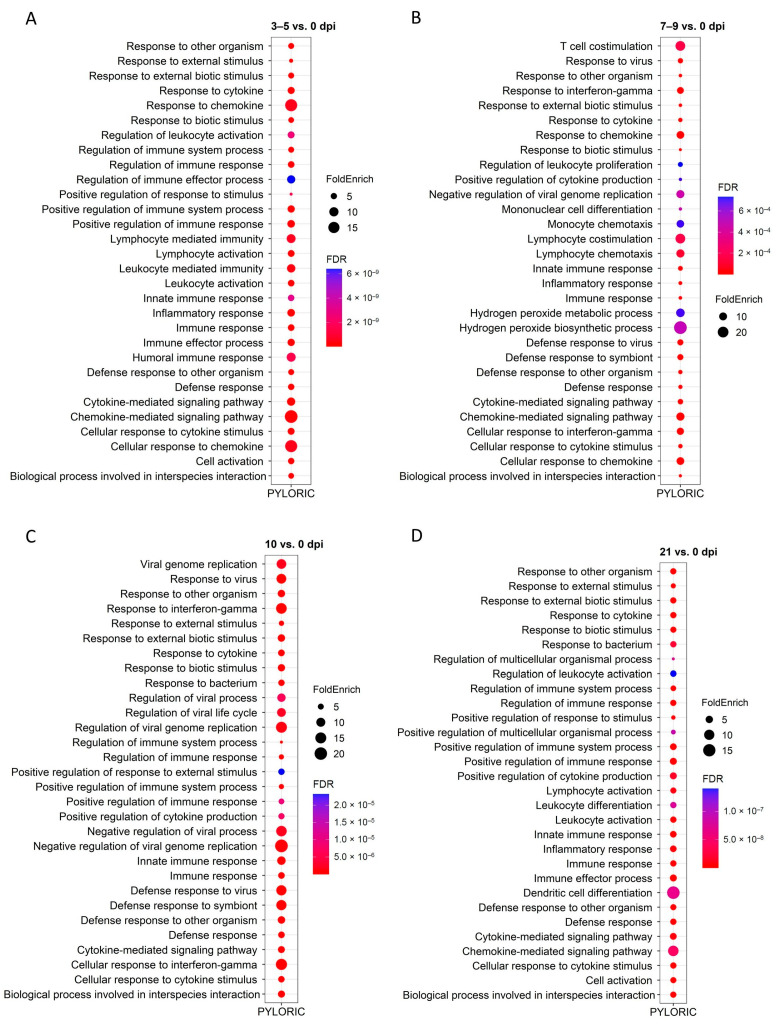
Top 30 enriched GO pathways for differentially expressed genes (DEGs) of cattle pyloric (PYL) mucosal tissue at four time points (3–5, 7–9, 10, and 21 dpi) when compared to 0 dpi (control). (**A**), 3-5 vs. 0 dpi; (**B**), 7–9 vs. 0 dpi; (**C**), 10 vs. 0 dpi; (**D**), 21 vs. 0 dpi. FoldEnrich: Fold Enrichment.

To better understand their biological functions, the enrichment of DEGs was tested at each time point and tissue for terms in the GO and KEGG databases using ShinyGO [16]. Figure 5 and Figure 6 show the top 30 GO-enriched BP. Infection induced differential expression of genes related to immune responses in both tissues and all time point comparisons. In FUN at 21 dpi, 25 of the top 30 GO processes are related to immune responses (Figure 5). In PYL, greater than 70% of the top 30 GO terms are related to defense at all time points (Figure 6). The GO terms related to defense included immune response, defense response, response to cytokine, inflammatory response, cytokine-mediated signaling pathway, cellular response to cytokine stimulus, positive regulation of cytokine production, chemokine-mediated signaling pathway, and others. Of all the significant GO-enriched terms for each tissue and time point (Table S7), slightly more enriched GO terms were identified for 21 vs. 0 dpi comparison in FUN than in PYL. In addition, PYL had more enriched terms when compared to FUN for 3–5 vs. 0 dpi and 10 vs. 0 dpi comparisons. In FUN, immune/defense-related GO terms represent ~35%, ~53%, ~44%, and ~59% of the GO terms at 3–5 dpi, 7–9 dpi, 10 dpi, and 21 dpi, respectively (Table S7). In PYL, immune-related GO terms represent ~52%, ~47%, ~46%, and ~53% of the GO terms at 3–5 dpi, 7–9 dpi, 10 dpi, and 21 dpi, respectively (Table S7). Also, a higher proportion of immune-related terms were present at 21 dpi in both mucosal tissues. In FUN at 21 dpi, a total of 238 DEGs are identified in the 214 pathways, which include genes encoding interleukins (*IL10*, *IL13*, *IL26*, *IL1A*, *IL1B*), IRF4, interferon-induced proteins (*IFIT2*, *IFIT3*, *IFI44*, and *IFI44L*), C-X-C motif chemokine ligands and receptors (*CXCL8*, *CXCL11*, *CXCR1*, and *CXCR2*), *EGR3*, *FCRL3*, *CDs* (*CD27*, *CD48*, *CD84*), MMPs (*MMP9*, *MMP12*, and *MMP14*), TNFSFs (*TNFSF8*, *TNFSF8*, *TNFSF11*, and *TNFSF15*), C2/3, HHLA2, PTGER2, and LTF (Table S7). In PYL at 21 dpi, a total of 133 DEGs are identified in the 181 pathways, including genes encoding interleukins (*IL10*, *IL13*, *IL1B*, *IL7R*), C-C motif chemokine ligands (*CCL1*, *CCL3*, *CCL4*, *CCL8*, etc.), C-X-C motif chemokine ligands and receptors (*CXCL8*, *CXCL10*, *CXCL11*, *CXCR1*, and *CXCR2*), CDs (*CD4*, *CD27*, *CD40*, CD101, etc.), MMPs (*MMP9*, *and MMP12*), IRF4, GZMA/B, TNFRSFs (*TNFRSF17*, and *TNFRSF13B*), C2/3, HHLA2, and PTGDR (Table S7).

Other than the immune-related GO terms described above, enriched GO terms are also related to nutrient utilization, such as lipid metabolism and transport, cell activation and migration, collagen catabolic process, ERK1 and ERK2 cascade, and regulation of hormone levels (Table S7).

KEGG enrichment in FUN showed that nematode infection affected cytokine-cytokine receptor interactions, IL-17 signaling pathway, and chemokine signaling pathway in all four time points (Figure S2, Table S8). KEGG enrichment in PYL also showed that the nematode infection affected immune responses involving IL-17 or chemokine signaling pathways, cytokine-cytokine receptor interaction, and intestinal immune network for IgA production (Figure S3, Table S8). The infection also affected pathways involved in various diseases (Figures S2 and S3, Table S8).

### 2.5. Gene Co-Expression

**Figure 7:**
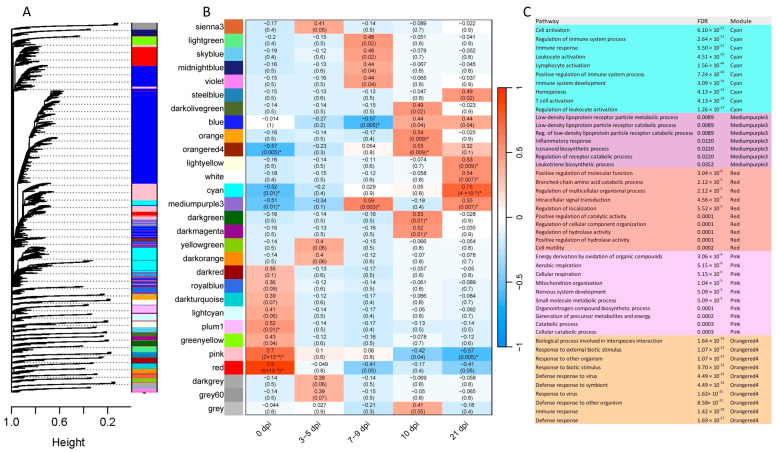
Weighted gene co-expression network analysis (WGCNA) for fundic (FUN) mucosal tissue at different time points. (**A**) Clustering dendrogram of genes with assigned merged module colors. (**B**) Heatmap of module–trait relationships depicting correlations between module eigengenes and traits representing infection stages. Numbers in parentheses in the table represent the correlation between *r* and *p*-values. The degree of correlation is shown with the color legend. (**C**) GO-enriched terms in the significant modules. * The modules with a *p*-value of ≤0.05 and a correlation coefficient of ≥0.5 are considered candidate modules.

**Figure 8:**
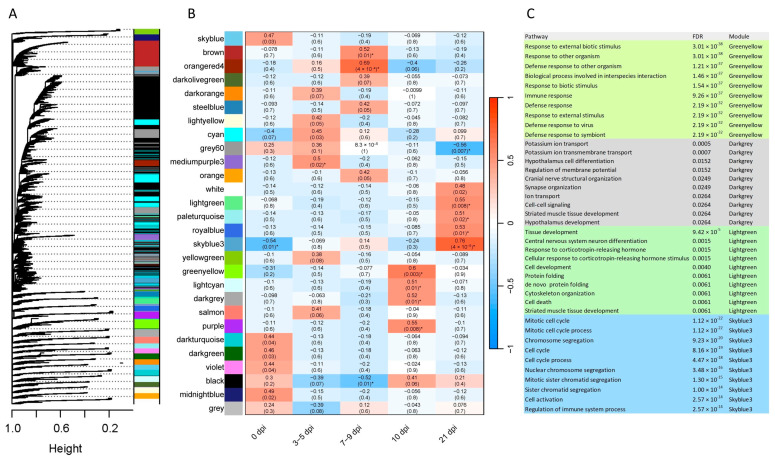
Weighted gene co-expression network analysis (WGCNA) for pyloric (PYL) mucosal tissue at different time points. (**A**) Clustering dendrogram of genes with assigned merged module colors. (**B**) Heatmap of module–trait relationships depicting correlations between module eigengenes and traits for infection stages. Numbers in parentheses in the table represent the correlation between the *r* and the *p*-value. The degree of correlation is shown with the color legend. (**C**) GO-enriched terms in the significant modules. * The modules with a *p*-value of ≤0.05 and a correlation coefficient of ≥0.5 are considered candidate modules.

Co-expression analysis for gene networks in each tissue at different time points was performed with the WGCNA R package [17]. For FUN and PYL, 29 and 28 co-expression modules were obtained, respectively (Figure 7 and Figure 8). The module–trait relationships were additionally analyzed using each time point as a factor of interest (*x*-axis) to correlate to each module (*y*-axis). For FUN, 0, 7–9, 10, and 21 dpi were significantly correlated (*p*-value ≤ 0.05, correlation ≥ 0.5) with 6, 2, 4, and 5 modules, respectively (Figure 7). For PYL, 0, 3–5, 7–9, 10, and 21 dpi were significantly correlated (*p*-value ≤ 0.05, correlation ≥ 0.5) with 1, 1, 3, 4, and 5 modules, respectively (Figure 8). GO enrichment analysis of the significant modules showed immune-related processes in FUN (cyan, mediumpurple3, and orangered4 modules), such as immune/defense/inflammatory response, immune system development, leukocyte/T cell/lymphocyte activation, and leukotriene biosynthetic process (Figure 7C). Similarly, in PYL, immune-related processes (greenyellow and skyblue3 modules) such as regulation of immune system process, immune/defense response, and defense response to other organisms were detected (Figure 8C). Other nutrition-related biological processes were also enriched in the observed modules, which include intracellular signal transduction (FUN), cell motility (FUN), cellular catabolic process (FUN), cell/tissue development (PYL), cell cycle and activation (PYL), and cell–cell signaling (PYL) (Figure 7C and Figure 8C).

### 2.6. Identification of Networks and Canonical Pathways Using IPA

DEGs from FUN and PYL of each time point were used for IPA analysis [18]. For FUN, a total of 62 regulatory networks with an IPA score of ≥10 were identified for all time points (Table S9). Many of these FUN networks characterize immune functions or GI-related diseases, particularly at 21 dpi, where 11 out of 62 immune-related networks were identified. For PYL, a total of 49 networks were identified at all time points (Table S9). Several immune-related networks were also identified in this tissue, such as inflammatory response, immunological disease, and immune cell trafficking.

Enriched canonical pathways for the two tissues and time points were also obtained. The complete list of enriched canonical pathways for all tissues is summarized in Table S10. The top 6 enriched canonical pathways for each time point and respective tissues are shown in Figure S4. Most of the top canonical pathways were related to immune responses in all time points and tissues, particularly at 21 dpi. Some of the pathways having a z score of ≥2, which is indicative of an activation state, also include the pathogen-induced cytokine storm signaling pathway in FUN at 21 dpi (z score = 5.88).

## 3. Discussion

### 3.1. Global Abomasal Responses to O. ostertagi Infection

Global abomasal responses to parasitic nematode infections in cattle have been poorly understood [19,20,21]. Here, using transcriptomic sequencing, differential expression of genes in two regions of the abomasum (fundic mucosa (FUN) and pyloric mucosa (PYL)) were investigated to understand the genes and pathways involved in host responses to infections with *O. ostertagi*. Host responses were studied across a time course that covered all parasitic stages of the parasite’s life cycle. Responses were different for each time point and in both FUN and PYL. The largest changes in DEGs occurred between 7–9 and 10 dpi in both tissues, corresponding to the transition from late-stage L4 to young adult worms. Many of the observed DEGs are involved in host immune responses. Pathway analyses revealed many significantly affected immunological gene networks, especially at 21 dpi, when inflammatory responses peaked. This study showed that there was a significant increase in checkpoint genes and those relating to T cell exhaustion and a lack of upregulated epithelium-derived alarmins such as IL-33, IL-25, and TSLP. These data may explain why the infection triggers not just Th2 responses, which are required for protection against helminth infection in the murine model, but also those of Th1 and Th17, consistent with a mixed response linked to immune evasion by the parasite. The data presented here strongly indicate that *O. ostertagi* infection initiates robust host immune responses while concurrently inducing immune interference and/or diversion. This study is the first study to investigate transcriptomic changes in abomasal mucosa in response to early *O. ostertagi* infection in Holstein cattle and identify consistent and nuanced temporal differences in gene expression in these two mucosal tissue types.

Infection induced reproducible and evolving changes in FUN and PYL. At each of the time points, fewer than 50% of the DEGs were shared among tissue types, suggesting a clear difference between the responses in each. More DEGs were detected in FUN than in PYL, consistent with previous reports that *O. ostertagi* predominantly resides in fundic gastric glands [22]. If so, early responses (prior to 10 dpi) in the PYL are more likely the result of secreted parasite products than direct interactions between the worms and the tissues. Relatively few DEGs occurred as early as 3–5 dpi, the initial period when the worm was at the late L3 and early L4 stages. More DEGs were observed in both FUN and PYL following the transition to L4, possibly in response to changes in parasite excretory/secretory (ES) products that interact with host tissues at this life stage. Most DEGs occurred 21 days post-infection in the fundic tissue and 7–9 and 21 post-infection in the pyloric tissue. At 21 days, the adult worms are in the lumen of the abomasum and at their peak reproductive stage, possibly causing tissue damage while excreting new ES products from the mature worms [23]. The changes in gene expression seem to mirror the development of the parasitic worms in the gut.

### 3.2. Host Immune Response to Infection

Multiple up- or downregulated genes were identified relating to host immune responses. In FUN, the example of highly upregulated DEGs includes molecules involved in innate immune responses, inflammatory responses, activation of eosinophils, cell proliferation, and a variety of processes connected to viral infection. Collectins and ITLN2 have been shown to be important for innate immune responses [24,25,26]. Collectins play essential roles in host innate immune responses, and collectin *CL43* appears to be unique to the *Bovidae* [25,27]. Both *CL43* and *CL46* were highly expressed in the FUN and PYL, indicating that these genes may be important for immune responses to nematode infections. Intelectins (intestinal lectins) have been implicated in the innate immune response in various human diseases, including Crohn’s disease [26]. In cattle, *ITLN2* was identified as a marker for paratuberculosis and plays an important role in the innate immune response to infections [28]. In addition, a previous study in mice showed that ITLN2 may play a role in the innate immune response to parasite infection, as ITLN2, one of the most abundant proteins expressed in infected jejunal epithelium, was upregulated in response to infection by GI nematodes [29]. *ITLN2* is highly expressed in the bovine FUN and PYL indicating that this gene may be involved in the development of host responses and in shaping host immunity early during *Ostertagia* infection.

Inflammation is a key outcome of ostertagiasis. Matrix metalloproteinases (MMPs), cadherins, and CLCA1 were all upregulated in this study. MMPs belong to a family of proteinases that possess multiple roles in immune responses, mediating inflammation and cellular reorganization and distribution [30,31,32]. The MMP3 protein functions to disrupt the barrier between endothelial cells and the nervous system and reduces leukocyte infiltration [30,33]. MMP12 has been shown to be involved in inflammatory processes and in macrophage migration [34,35,36]. High expression of multiple *MMP* gene members in FUN and PYL suggests that this gene family is essential to host immune responses to the nematode infection. Cadherins mediate several important processes in inflammation, such as cell adhesion, migration, and differentiation [37,38,39]. Interestingly, *CDH26* is highly expressed in human allergic GI tissue [37]. In mucus hypersecretory-related GI diseases, CLCA1 participates in the pathogenesis of colon colitis, ulcerative colitis, and GI parasite infections [40]. The *CLCA1* gene was upregulated in FUN and PYL, especially in FUN at 21 dpi, consistent with being one of a collection of inflammation-related genes elicited by nematode infection.

Eosinophils play a key role in host resistance to GI nematode infections, characterized by expression of eotaxin or C-C chemokine receptor 3 (CCR3), proteoglycan 3 (PRG3), and eosinophil peroxidase (EPX) [41]. Our study identified upregulation of both *PRG3* and *CCR3* in FUN, while in PYL *PRG3* was upregulated only at 3–5 dpi, suggesting a differential and physiological role of FUN and PYL during the infection. As noted above, at 3–5 dpi, responses in the PYL are likely due to distal parasite products rather than the worms themselves because of the dearth of chief and parietal cells in the gastric glands of the PYL. A study with mice infected with GI nematodes showed CCR3 playing an important role in eosinophil recruitment in the intestine [42]. Another study demonstrated the role of CCR3 in facilitating eosinophil recruitment [43].

Cell proliferation plays a role in hyperplasia during *O. ostertagi* infection. Transcobalamin I (TCN1) transports and protects cobalamin (vitamin B12) from the acidic fluid in the stomach by forming a TCN1-B12 complex [44]. TCN1 also has important roles in cell proliferation and has been shown to be highly expressed by colon cancer cells and proposed as a negative prognostic biomarker [45]. *TCN1* was highly expressed in FUN and PYL, indicating that this gene indeed plays a role during *O. ostertagi* infection, possibly by contributing to cellular hyperplasia [46].

### 3.3. Viral Infection Responses Induced by O. ostertagi

Interestingly, our study found that *O. ostertagi* induced the expression of genes that were also associated with host responses to viral infections. These include *LRRC15*, which was upregulated in both FUN and PYL. In fact, this gene is a therapeutic target for cancer [47] and can function as a receptor for SARS-CoV-2 spike protein, thereby controlling viral load and regulating antiviral transcriptional programs [48]. Additionally, *ISG15*, an interferon-induced protein, was also upregulated in FUN and PYL, suggesting that the antiviral responses were activated either directly by *O. ostertagi* infection or by opportunistic viruses in the environment [49]. Indeed, type I interferons play an essential role in the initiation of immunity [50] where data have been presented demonstrating a significant upregulation of IFN-γ during bolus infections with *O. ostertagi* [51]; however, the mechanisms by which type interferons and antiviral responses are involved during nematode infection remain unclear. Overall, FUN and PYL respond to *O. ostertagi* dynamically in a time-dependent manner by expressing shared and unique DEGs with an overall dramatic elevation of immune-related genes toward 21 dpi.

Notably, GO and KEGG enrichment analysis of DEGs revealed pathways relating to immune/defense responses at all time points, especially at 21 dpi, which may represent the peak of the nematode-elicited inflammation during the first few weeks of infection. This is supported by the worms controlling local inflammatory responses while in the gastric glands (<10 dpi). Pathways such as responses to cytokines, cytokine-mediated signaling pathways, inflammatory responses, and lymphocyte/leukocyte activation are among the most robust upregulated responses. Similarly, at least 21 interleukin genes were upregulated in both tissues, especially at 21 dpi in FUN. Previous studies profiled a subset of cytokine genes induced in cattle infected with *O. ostertagi* [51]. In general, GI nematode infections in laboratory models result in high levels of Th2 cytokines such as IL-4, IL-5, IL-6, and IL-13 [52]. In the present study, *IL4*, *IL4I1,* and *IL6R* were upregulated only in FUN, and *IL13*, *IL13RA2,* and *IL5RA* were upregulated in both FUN and PYL. Similarly, the interferon-gamma (*IFNG*) gene and *IL1B*, both Th1 cytokine genes, were also upregulated in both FUN and PYL. Th17 genes were also upregulated in FUN and PYL, such as *IL17REL*, *IL17F*, *IL17RB*, *IL22RA1,* and *IL22RA2*. A mixed Th response characterized by Th1, Th2, and Th17 cytokine genes was observed in this study in response to the nematode infection, corroborating with previous studies in cattle [4,52,53].

The *WC1* gene is present in some animal species, such as cattle, sheep, and swine, but is not present in humans. A previous study demonstrated the existence of 13 members of the *WC1* gene family in the bovine [54]. WC1 is a transmembrane glycoprotein and is uniquely expressed on gamma/delta T cells, which are essential for the initial response to inflammation and infection [55]. In this study, the *WC1* gene was upregulated at 21 dpi in both FUN and PYL and at 10 dpi in PYL, whereas *WC1-10* was upregulated at 21 dpi in both FUN and PYL. These results suggest that gamma/delta T cells may also be involved in immunity to nematode infections. The *IL4I1* gene is induced by IL-4 in B cells and also expressed by macrophages and dendritic cells. It is present in enriched GO terms and may play a role in immune evasion by regulation of adaptive immune response, leukocyte proliferation and differentiation, and T cell-mediated immunity [56]. *IFNG* is also involved in several enriched GO terms, such as positive regulation of IL-6 production, chemokine production, and regulation of T cell differentiation, implicating an essential role of this key Th1 cytokine gene during *Ostertagia* infection. IFNG is a critical cytokine in both innate and adaptive immunity [57]. The gene *FCER1A* (IgE receptor) was upregulated in FUN (7–9 and 21 dpi) in our study. In a previous study in cattle infected with *O. ostertagi* [57], this IgE receptor gene was proposed as an important element in the immunity to *O. ostertagi*. The *FCER1A* gene was shown to be involved in two enriched GO terms, immune response, and defense response; KEGG pathway analysis confirms its involvement in the Fc epsilon RI signaling pathway. The gene encoding granzyme B (*GZMB*) was also reported to be important for immunity in cattle infected with *O. ostertagi* [58]. In our study, *GZMB* was upregulated in both FUN and PYL. *GZMB* supports several enriched GO terms, such as innate immune response, immune effector process, and leukocyte-mediated immunity. In addition, several IPA networks related to immune defense were upregulated at all time points in FUN and PYL, which include inflammatory disease or response, cell-mediated immune response, and immune cell trafficking. Immunological pathways were particularly enriched in IPA networks and canonical pathways, further confirming our GO and KEGG analyses.

### 3.4. Co-Expression Analysis and Immune-Related Processes

GO enrichment analysis of the significant co-expression module genes showed several immune-related processes in FUN and PYL, particularly at 21 dpi. These clusters of genes may share similar functions; if so, they implicate biological processes as having special importance. For example, in FUN, a module (cyan) positively correlated with 21 dpi was identified as containing genes of immune-related functions. The co-expression results corroborate the additional downstream analyses showing several highly expressed, immune-related genes during an *Ostertagia* infection.

Tuft cells play a key role in mucosal immunity to GI nematode infections [59]. Here, infection with *O. ostertagi* induced the expression of some Tuft cell markers, including *POU2F3*, *CHAT*, *TRPM5*, and *GFI1B* in cattle. Yet, no significant changes were observed in other typical Tuft/epithelial cell genes and alarmin genes, such as *DCLK1*, *IL-25*, *IL-33*, *TSLP*, and *CK18*. These results indicate that Tuft/epithelial cell response to mucosal pathogens such as GI nematodes may vary among host species or that bovine nematodes downregulate Tuft cell-derived alarmin genes and some of the Tuft cell markers.

T cell exhaustion can impair host responses to pathogens [60,61]. Elevated expression of several typical T cell exhaustion genes was identified in both tissues (such as *CD274*, *CTLA4*, *BATF2*, *ICOS*, *IRF4*, *LAG3*, *PDCD1LG2*, *PLA2G5*, *PTPN7*, and *TIGIT*). Possible T cell exhaustion induced by *O. ostertagi* may help explain why immunity to *Ostertagia* infection develops slowly, and repeated exposure to this parasite does not induce robust protective immunity.

## 4. Materials and Methods

### 4.1. Experimental Animals

**Figure 9:**
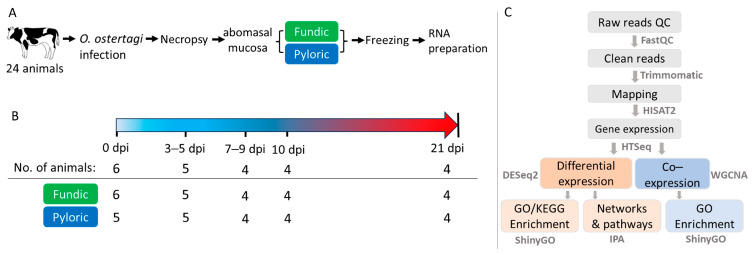
Experimental design. (**A**) Experimental flow chart. (**B**) Infection timeline and number of animals and tissues at each time point. (**C**) Data analysis workflow. Samples from 3 and 5 dpi (3–5 dpi) and those from 7 and 9 dpi (7–9 dpi) were combined to increase the sample size per time point based on PCA analysis.

Holstein steers used in this study were from the Beltsville Agricultural Research Center (BARC) Dairy Unit and raised helminth-free from birth. All animals were weaned at 3 months of age and had free access to water and feed. During infection, animals remained free access to water and hay and were supplemented with approximately 5 lb of the Feedlot Pellet feed per head per day (Farmers Cooperative Association, Inc., Frederick, MD, USA). We used an *O. ostertagi* isolate, which was obtained in Beltsville MD and has been maintained for research for decades in BARC. *O. ostertagi* was propagated in helminth-free calves as described previously [62] (Figure 9). All animals (n = 24, approximately 5 months of age) received either a single, oral bolus infection with *O. ostertagi* L3 (200,000 per animal), or PBS as the control on day 0. Animals were then euthanized on day 0 post-infection (0 dpi, uninfected controls; n = 6), 3 (n = 3), 5 (n = 3), 7 (n = 2), 9 (n = 2), 10 (n = 4) or 21 (n = 4) dpi. At necropsy, abomasal fundic (FUN) and pyloric (PYL) mucosa were carefully dissected from *muscularis externa*, snap-frozen in liquid nitrogen, and stored at −80 °C until processed. All animals were euthanized by captive bolt pistol stunning, followed immediately by exsanguination. Animal care and use were approved by BARC IACUC (protocol number 19-012).

### 4.2. RNA Isolation, Library Construction, and Sequencing

Frozen tissues were pulverized while submerged in liquid nitrogen using a cryogenic grinder (SPEX SAMPLEPREP, Metuchen, NJ, USA). Total RNA from each sample was extracted using the TRIzol^®^ Reagent (Thermo Fisher, Waltham, MA, USA). Purified RNA was resuspended in nuclease-free water and stored at −80 °C until used. RNA quality was assessed using Agilent Bioanalyzer 2100 (Santa Clara, CA, USA), and all samples with a RIN (RNA Integrity) of 6 or greater were submitted for RNA sequencing by Novogene (Sacramento, CA, USA) and by Azenta/Genewiz (South Plainfield, NJ, USA) on an Illumina HiSeq 4000 sequencing machine which generated paired-end 150 bp reads.

### 4.3. Pre-Processing Sequence Data and Alignment

All raw reads were quality-tested with FastQC (version 0.11.9) (http://www.bioinformatics.babraham.ac.uk/projects/fastqc/; accessed on 1 June 2023). Clean reads were obtained by removing adaptors, and low-quality reads were obtained with Trimmomatic (version 0.38) [63] using the following parameters: TruSeq3-PE.fa:2:30:10, LEADING:3, TRAILING:3, SLIDINGWINDOW:4:15 and MINLEN:36. The reads were then mapped to the cattle ARS-UCD1.3 reference genome available at the time [14] using HISAT2 (version 2.2.1) [64]. BAM files were generated after the mapping and sorted using SAMtools (version 1.9) [65].

### 4.4. Gene Expression and Differential Expression Analysis

HTSeq (version 2.0.2) was used to obtain the gene counts directly from the BAM alignment files using the HTSeq-count function [66]. Genes with zero counts and those located on mitochondrial and unplaced chromosomes were removed. The normalized counts were obtained with DESeq2 (version 1.38.3) [15]. DESeq2 normalization utilizes the median of the ratios of observed counts to calculate size factors [67]. Then, principal component analysis (PCA) using the gplots package from R (version 4.2.1) was utilized to assess sample quality and identify potential outliers and batch effects from the normalized counts. Since there were too few animals on 3, 5, 7, or 9 dpi, the time points were combined into 3–5 dpi and 7–9 dpi based on their similarities, as indicated by PCA shown in Figure 1.

Differential expression analyses for each tissue were performed with DESeq2 (version 1.38.3) [15] using unnormalized gene counts generated by HTSeq to identify DEGs based on dpi with 0 dpi as control (e.g., 0 dpi vs. 3–5, 7–9, 10 or 21 dpi) or between two neighboring time points (0 vs. 3–5 dpi, 3–5 vs. 7–9 dpi, 7–9 vs. 10 dpi, and 10 vs. 21 dpi). The DEGs were defined as those with an FDR < 0.05 and |log_2_FC| > 2. DEGs with an FDR < 0.05 were used for downstream analyses to identify Gene Ontology (GO) and Kyoto Encyclopedia of Genes and Genomes (KEGG) enriched terms or pathways using ShinyGO (version 0.77) [16]. QIAGEN Ingenuity Pathway Analysis (IPA) (version 94302991) [18] was used to identify signaling and metabolic pathways of genes with relevant biological functions in fundic and pyloric DEGs at different time points. As input, DEGs with log fold changes and FDR were imported and mapped to their corresponding annotations. The “Core Analysis/expression analysis” function with default parameters, including networks with a maximum of 35 molecules and molecules of experimentally observed confidence, was used. *Homo sapien* was chosen for the species option. The expression in fold change was used to calculate z scores in the core analysis. Networks with scores of ≥10 were deemed as significant. The enriched canonical pathways were identified by applying a -log (*p*-value) threshold of >2 or a *p*-value of <0.01 and z scores. Z scores greater than or equal to 2 represent predictions of activation, while z scores less than or equal to −2 are indicative of predictions for inhibition.

### 4.5. Co-Expression Analysis

The co-expression modules for PYL and FUN mucosa were generated using the WGCNA (version 1.72-1) [17]. All genes (normalized counts from 25,316 genes) for each tissue were utilized to generate the co-expressed functional gene modules. The topological overlap matrix (TOM) was constructed with a soft-thresholding power of 8. Then, the modules were identified using the dynamic tree cut method with a minimum size of 30. For module grouping, a threshold of 0.3 was employed, corresponding to a correlation of 0.7. The module–trait relationships were obtained for each tissue type, and modules with a *p*-value of ≤0.05 and a correlation coefficient of ≥0.5 were considered as the candidate modules. The genes from each significant module were used for the GO enrichment analysis using ShinyGO (version 0.77) [16] with an FDR of <0.05 for GO biological processes (BP) terms.

## 5. Conclusions

These data significantly advance our understanding of how cattle respond to nematode infections by elucidating time-dependent and tissue-specific transcriptional responses of genes and networks. Studying gene expression circumvents restrictions imposed by the deficit of immunologic reagents for cattle (when compared to human and rodent models). Our data demonstrate the progressive development of host responses to infection by developing stages of *O. ostertagi*, and common and unique aspects of bovine host immune responses in different regions of the abomasum, which do not neatly support Th1, Th2, nor Th17 responses. Instead, *O. ostertagi* infection elicits a mixed response, characterized by a multitude of Th1, Th2, and Th17 “specific” cytokine genes and many other genes of unknown function. Clearly, bovine immune responses to the GI nematode are very different from those demonstrated in the murine models. Further, the bovine nematodes may be equipped with powerful yet unique weapons fostering ultimate immune evasion, which may be supported by elevated expression of T cell exhaustion and immune checkpoint genes in *Ostertagia*-infected animals. Further data mining of these datasets from a nutritional perspective using new bioinformatic technologies will reveal mechanisms and pathways specific to the ruminant host and parasite interactions. Finally, these data have been generated using bolus infections, which produce worms that develop synchronically within the host. This was done to enable gene expression profiling related to the time course of infection and worm development. An interesting corollary to this study would be to use low-level, trickle infections to mimic natural conditions and help reveal the presence or absence of pathways necessary for protection.

## Data Availability

The RNA-seq data were deposited in the NCBI Sequence Read Archive (SRA) under the accession number PRJNA994089.

## Supplementary Materials

The supporting information can be downloaded at: https://www.mdpi.com/article/10.3390/ijms26052264/s1.

## Abbreviations

BARC	Beltsville Agricultural Research Center
BP	biological processes
CCL or CXCL	C-C or C-X-C motif chemokine ligand
CCR or CXCR	C-C or C-X-C motif chemokine receptor
DEGs	differentially expressed genes
DPI	days post-infection
EPX	eosinophil peroxidase
ES	excretory/secretory
FUN	abomasal fundic mucosa
GI	gastrointestinal
GZMB	granzyme B
GO	gene ontology
IFNG	interferon-gamma
Ig	immunoglobulin
IL	interleukin
IPA	Ingenuity Pathway Analysis
KEGG	Kyoto Encyclopedia of Genes and Genomes
L3	stage 3 larvae
MMPs	matrix metalloproteinases
PBMCs	peripheral blood monocytes
PCA	principal component analysis
PCR	polymerase chain reaction
PYL	abomasal pyloric mucosa
PRG3	proteoglycan 3
RNA-seq	RNA sequencing
SRA	Sequence Read Archive
TCE	T cell exhaustion
TCN1	Transcobalamin I
Th	T helper
TOM	topological overlap matrix
WGCNA	weighted gene co-expression network analysis
