# Supplementary material for: Transcriptional Profiling of Abomasal Mucosa from Young Calves Experimentally Infected with Ostertagia ostertagi
Source: Int J Mol Sci. 2025 Mar 4;26(5):2264. doi: 10.3390/ijms26052264 (PMC11900041; doi:10.3390/ijms26052264)
Supplement: Supplementary file 1 [file ijms-26-02264-s001.zip › Figures and Tables captions.pdf]

Supplementary Figures

Figure S1. Volcano plots of differentially expressed genes (DEGs) of cattle fundic (FUN) and pyloric (PYL) mucosal tissues. (A) Four time points (3-5, 7-9, 10, and 21 dpi) *vs*. 0 dpi (control) comparisons. (B) Neighboring time point comparisons (0, 3-5, 7-9, 10, and 21 dpi). Each dot represents an individual gene. Black dots represent the genes that were not significantly expressed, while the green dots indicate the genes that were significantly upregulated or downregulated (FDR < 0.05 and |log2FC| >2).

Figure S2. Top 30 enriched KEGG pathways for differentially expressed genes (DEGs) of cattle fundic mucosal tissue (FUN) at four time points (3-5, 7-9, 10, and 21 dpi) when compared to 0 dpi (control). FoldEnrich: Fold Enrichment. Gene count is the number of genes enriched in a KEGG pathway.

Figure S3. Top 30 enriched KEGG pathways for differentially expressed genes (DEGs) of cattle pyloric mucosal tissue (PYL) at four time points (3-5, 7-9, 10, and 21 dpi) comparing to 0 dpi (control). FoldEnrich: Fold Enrichment. Gene count is the number of genes enriched in a KEGG pathway.

Figure S4. Top six canonical pathways identified by Ingenuity for the DEGs in fundic (FUN) and pyloric (PYL) mucosa. The y-axis displays the canonical pathways at each time point for fundic and pyloric tissues. The x-axis displays the –log of the Benjamini–Hochberg corrected *p-value*. The red line indicates the threshold at a Benjamini–Hochberg corrected p < 0.01. The four time points are represented by different colored bars.

Supplementary Tables

Table S1. Mapping summary statistics of 45 samples from cattle fundic (FUN) and pyloric (PYL) mucosal tissues.

Table S2. The number of differentially expressed genes (DEGs) at four time points (3-5, 7-9, 10, and 21 dpi) when compared to 0 dpi (control) in cattle fundic (FUN) and pyloric (PYL) mucosal tissues.

Table S3. The number of differentially expressed genes (DEGs) at neighboring time points (0 dpi *vs*. 3-5 dpi, 3-5 dpi *vs*. 7-9 dpi, 7-9 dpi *vs*. 10 dpi, and 10 dpi *vs*. 21 dpi) in cattle fundic (FUN) and pyloric (PYL) mucosal tissues.

Table S4. The shared and unique number of differentially expressed genes (DEGs) at four time points (3-5, 7-9, 10, and 21 dpi) compared to 0 dpi (control) in cattle fundic (FUN mucosal tissue.

Table S5. The shared and unique differentially expressed genes (DEGs) at four time points (3-5, 7-9, 10, and 21 dpi) when compared to 0 dpi (control) in cattle pyloric (PYL) mucosal tissue.

Table S6. Immune-related differentially expressed genes (DEGs) at four time points (3-5, 7-9, 10, and 21 dpi) when compared to 0 dpi in cattle fundic (FUN) and pyloric (PYL) mucosal tissues under 6 biological categories including T cell exhaustion (TCE), Th1, Th2, Th17, interleukin (IL), and Tuft cell genes with respective log2FC. Red highlights indicate upregulated genes and blue highlights represent downregulated genes (FDR < 0.05 and |log2FC| >2).

Table S7. Enriched GO pathways (FDR < 0.05) for differentially expressed genes (DEGs) of cattle fundic (FUN) and pyloric (PYL) mucosal tissues at four time points (3-5, 7-9, 10, and 21 dpi) when compared to 0 dpi (control).

Table S8. Enriched KEGG pathways (FDR < 0.05) for differentially expressed genes (DEGs) of cattle fundic (FUN) and pyloric (PYL) mucosal tissues at four time points (3-5, 7-9, 10, and 21 dpi) comparing to 0 dpi (control).

Table S9. Regulatory networks (IPA score ≥ 10) for differentially expressed genes (DEGs) of cattle fundic (FUN) and pyloric (PYL) mucosal tissues at four time points (3-5, 7-9, 10, and 21 dpi) when compared to 0 dpi (control).

Table S10. Enriched canonical pathways (*p*-value < 0.01) for differentially expressed genes (DEGs) of cattle fundic (FUN) and pyloric (PYL) mucosal tissues at four time points (3-5, 7-9, 10, and 21 dpi) when compared to 0 dpi (control).
