# Supplementary material for: Transcriptional Profiling of Abomasal Mucosa from Young Calves Experimentally Infected with Ostertagia ostertagi
Source: Int J Mol Sci. 2025 Mar 4;26(5):2264. doi: 10.3390/ijms26052264 (PMC11900041; doi:10.3390/ijms26052264)
Supplement: Supplementary file 1 [file ijms-26-02264-s001.zip › Supp Figure All.pdf]

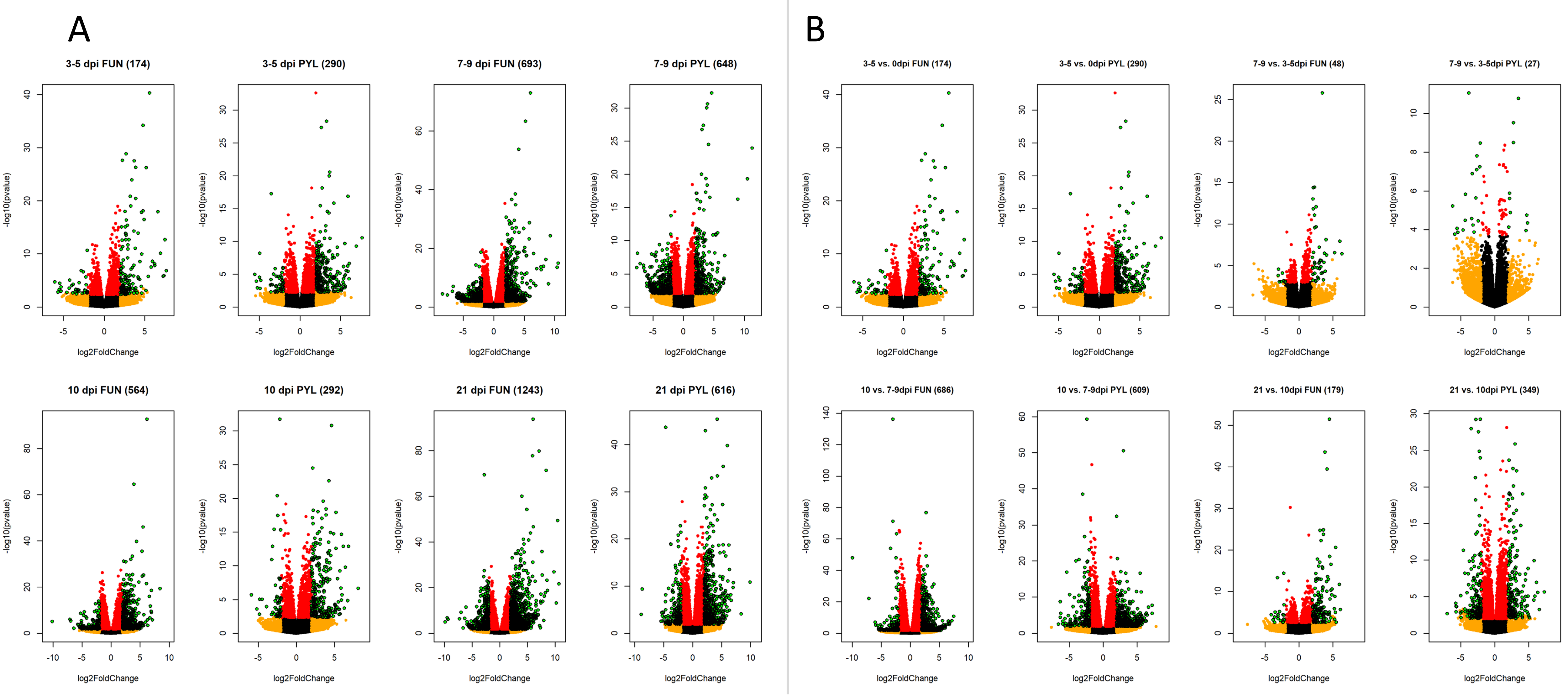
A
3-5 dpi FUN (174)
3-5 dpi PYL (290)
7-9 dpi FUN (693)
7-9 dpi PYL (648)
10 dpi FUN (564)
10 dpi PYL (292)
21 dpi FUN (1243)
21 dpi PYL (616)
B
3-5 vs. 0dpi FUN (174)
3-5 vs. 0dpi PYL (290)
7-9 vs. 3-5dpi FUN (48)
7-9 vs. 3-5dpi PYL (27)
10 vs. 7-9dpi FUN (686)
10 vs. 7-9dpi PYL (609)
21 vs. 10dpi FUN (179)
21 vs. 10dpi PYL (349)
-log10(pvalue)
log2FoldChange

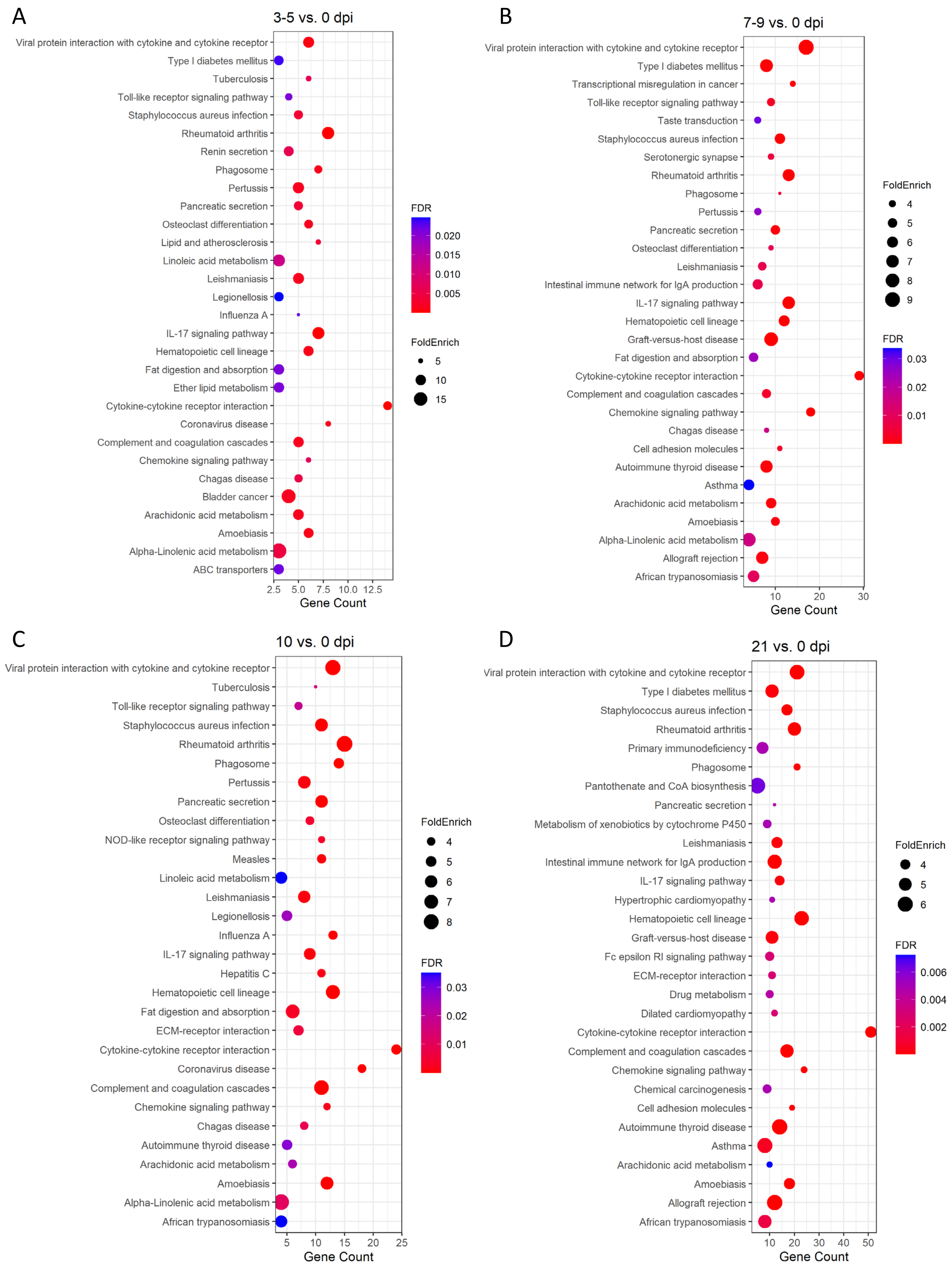

A
3-5 vs. 0 dpi
Viral protein interaction with cytokine and cytokine receptor
Type I diabetes mellitus
Tuberculosis
Toll-like receptor signaling pathway
Staphylococcus aureus infection
Rheumatoid arthritis
Renin secretion
Phagosome
Pertussis
Pancreatic secretion
Osteoclast differentiation
Lipid and atherosclerosis
Linoleic acid metabolism
Leishmaniasis
Legionellosis
Influenza A
IL-17 signaling pathway
Hematopoietic cell lineage
Fat digestion and absorption
Ether lipid metabolism
Cytokine-cytokine receptor interaction
Coronavirus disease
Complement and coagulation cascades
Chemokine signaling pathway
Chagas disease
Bladder cancer
Arachidonic acid metabolism
Amoebiasis
Alpha-Linolenic acid metabolism
ABC transporters
2.5 5.0 7.5 10.0 12.5
Gene Count
FDR
0.020
0.015
0.010
0.005
FoldEnrich
5
10
15
B
7-9 vs. 0 dpi
Viral protein interaction with cytokine and cytokine receptor
Type I diabetes mellitus
Transcriptional misregulation in cancer
Toll-like receptor signaling pathway
Taste transduction
Staphylococcus aureus infection
Serotonergic synapse
Rheumatoid arthritis
Phagosome
Pertussis
Pancreatic secretion
Osteoclast differentiation
Leishmaniasis
Intestinal immune network for IgA production
IL-17 signaling pathway
Hematopoietic cell lineage
Graft-versus-host disease
Fat digestion and absorption
Cytokine-cytokine receptor interaction
Complement and coagulation cascades
Chemokine signaling pathway
Chagas disease
Cell adhesion molecules
Autoimmune thyroid disease
Asthma
Arachidonic acid metabolism
Amoebiasis
Alpha-Linolenic acid metabolism
Allograft rejection
African trypanosomiasis
10 20 30
Gene Count
FoldEnrich
4
5
6
7
8
9
FDR
0.03
0.02
0.01
C
10 vs. 0 dpi
Viral protein interaction with cytokine and cytokine receptor
Tuberculosis
Toll-like receptor signaling pathway
Staphylococcus aureus infection
Rheumatoid arthritis
Phagosome
Pertussis
Pancreatic secretion
Osteoclast differentiation
NOD-like receptor signaling pathway
Measles
Linoleic acid metabolism
Leishmaniasis
Legionellosis
Influenza A
IL-17 signaling pathway
Hepatitis C
Hematopoietic cell lineage
Fat digestion and absorption
ECM-receptor interaction
Cytokine-cytokine receptor interaction
Coronavirus disease
Complement and coagulation cascades
Chemokine signaling pathway
Chagas disease
Autoimmune thyroid disease
Arachidonic acid metabolism
Amoebiasis
Alpha-Linolenic acid metabolism
African trypanosomiasis
5 10 15 20 25
Gene Count
FoldEnrich
4
5
6
7
8
FDR
0.03
0.02
0.01
D
21 vs. 0 dpi
Viral protein interaction with cytokine and cytokine receptor
Type I diabetes mellitus
Staphylococcus aureus infection
Rheumatoid arthritis
Primary immunodeficiency
Phagosome
Pantothenate and CoA biosynthesis
Pancreatic secretion
Metabolism of xenobiotics by cytochrome P450
Leishmaniasis
Intestinal immune network for IgA production
IL-17 signaling pathway
Hypertrophic cardiomyopathy
Hematopoietic cell lineage
Graft-versus-host disease
Fc epsilon RI signaling pathway
ECM-receptor interaction
Drug metabolism
Dilated cardiomyopathy
Cytokine-cytokine receptor interaction
Complement and coagulation cascades
Chemokine signaling pathway
Chemical carcinogenesis
Cell adhesion molecules
Autoimmune thyroid disease
Asthma
Arachidonic acid metabolism
Amoebiasis
Allograft rejection
African trypanosomiasis
10 20 30 40 50
Gene Count
FoldEnrich
4
5
6
FDR
0.006
0.004
0.002

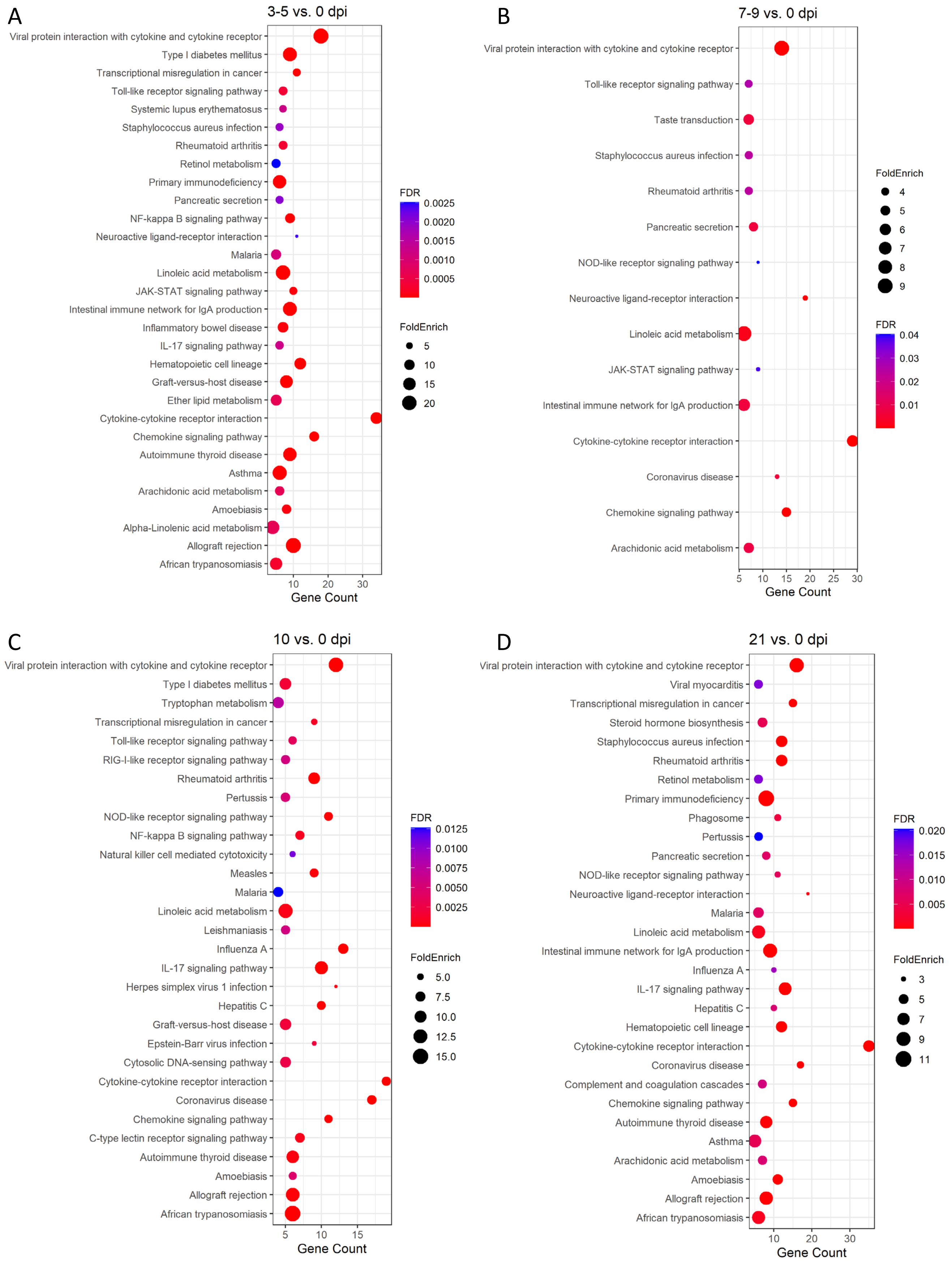

A
3-5 vs. 0 dpi
Viral protein interaction with cytokine and cytokine receptor
Type I diabetes mellitus
Transcriptional misregulation in cancer
Toll-like receptor signaling pathway
Systemic lupus erythematosus
Staphylococcus aureus infection
Rheumatoid arthritis
Retinol metabolism
Primary immunodeficiency
Pancreatic secretion
NF-kappa B signaling pathway
Neuroactive ligand-receptor interaction
Malaria
Linoleic acid metabolism
JAK-STAT signaling pathway
Intestinal immune network for IgA production
Inflammatory bowel disease
IL-17 signaling pathway
Hematopoietic cell lineage
Graft-versus-host disease
Ether lipid metabolism
Cytokine-cytokine receptor interaction
Chemokine signaling pathway
Autoimmune thyroid disease
Asthma
Arachidonic acid metabolism
Amoebiasis
Alpha-Linolenic acid metabolism
Allograft rejection
African trypanosomiasis
10 20 30
Gene Count
FDR
0.0025
0.0020
0.0015
0.0010
0.0005
FoldEnrich
5
10
15
20
B
7-9 vs. 0 dpi
Viral protein interaction with cytokine and cytokine receptor
Toll-like receptor signaling pathway
Taste transduction
Staphylococcus aureus infection
Rheumatoid arthritis
Pancreatic secretion
NOD-like receptor signaling pathway
Neuroactive ligand-receptor interaction
Linoleic acid metabolism
JAK-STAT signaling pathway
Intestinal immune network for IgA production
Cytokine-cytokine receptor interaction
Coronavirus disease
Chemokine signaling pathway
Arachidonic acid metabolism
5 10 15 20 25 30
Gene Count
FoldEnrich
4
5
6
7
8
9
FDR
0.04
0.03
0.02
0.01
C
10 vs. 0 dpi
Viral protein interaction with cytokine and cytokine receptor
Type I diabetes mellitus
Tryptophan metabolism
Transcriptional misregulation in cancer
Toll-like receptor signaling pathway
RIG-I-like receptor signaling pathway
Rheumatoid arthritis
Pertussis
NOD-like receptor signaling pathway
NF-kappa B signaling pathway
Natural killer cell mediated cytotoxicity
Measles
Malaria
Linoleic acid metabolism
Leishmaniasis
Influenza A
IL-17 signaling pathway
Herpes simplex virus 1 infection
Hepatitis C
Graft-versus-host disease
Epstein-Barr virus infection
Cytosolic DNA-sensing pathway
Cytokine-cytokine receptor interaction
Coronavirus disease
Chemokine signaling pathway
C-type lectin receptor signaling pathway
Autoimmune thyroid disease
Amoebiasis
Allograft rejection
African trypanosomiasis
5 10 15
Gene Count
FDR
0.0125
0.0100
0.0075
0.0050
0.0025
FoldEnrich
5.0
7.5
10.0
12.5
15.0
D
21 vs. 0 dpi
Viral protein interaction with cytokine and cytokine receptor
Viral myocarditis
Transcriptional misregulation in cancer
Steroid hormone biosynthesis
Staphylococcus aureus infection
Rheumatoid arthritis
Retinol metabolism
Primary immunodeficiency
Phagosome
Pertussis
Pancreatic secretion
NOD-like receptor signaling pathway
Neuroactive ligand-receptor interaction
Malaria
Linoleic acid metabolism
Intestinal immune network for IgA production
Influenza A
IL-17 signaling pathway
Hepatitis C
Hematopoietic cell lineage
Cytokine-cytokine receptor interaction
Coronavirus disease
Complement and coagulation cascades
Chemokine signaling pathway
Autoimmune thyroid disease
Asthma
Arachidonic acid metabolism
Amoebiasis
Allograft rejection
African trypanosomiasis
10 20 30
Gene Count
FDR
0.020
0.015
0.010
0.005
FoldEnrich
3
5
7
9
11

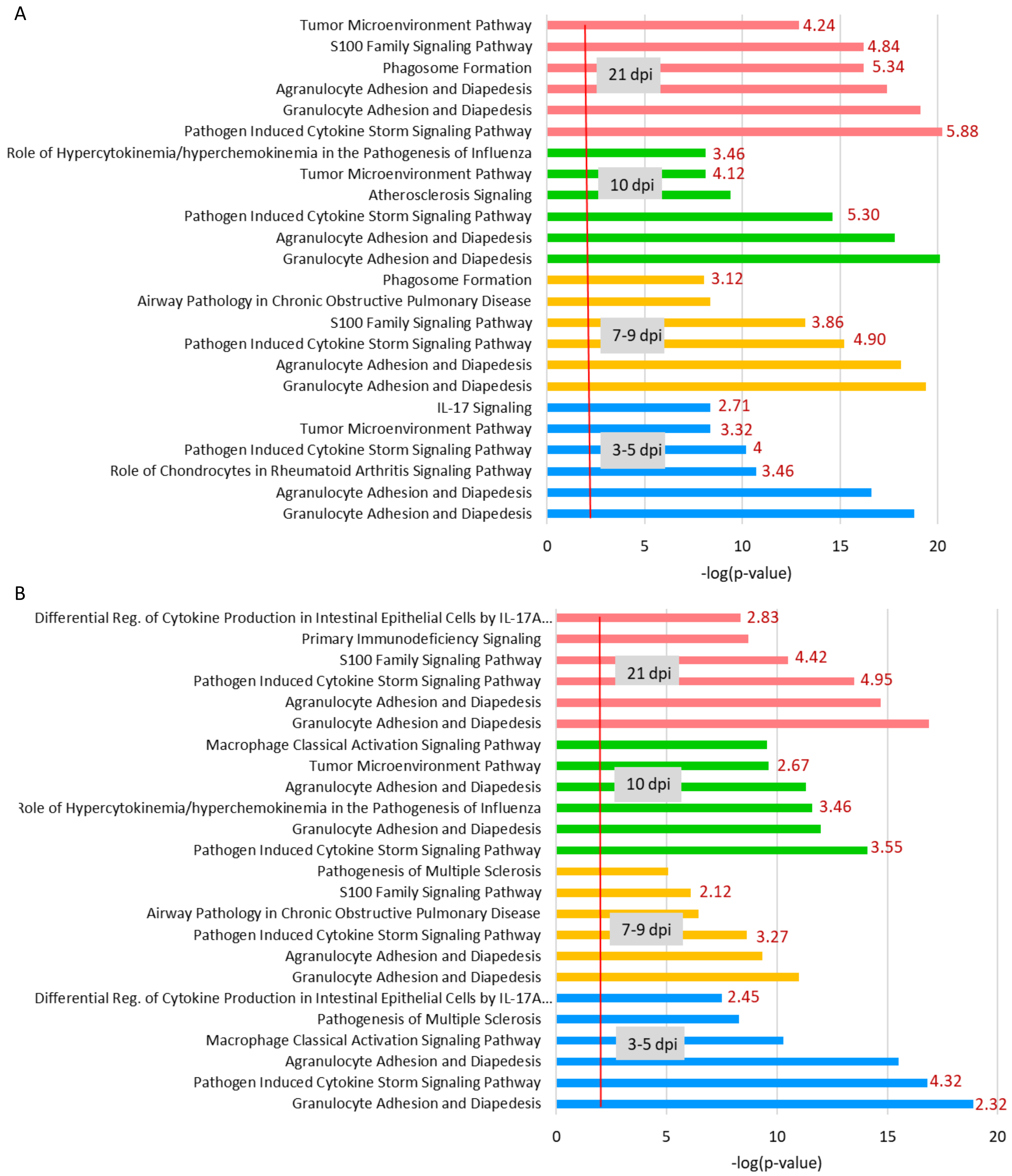
A
Tumor Microenvironment Pathway
S100 Family Signaling Pathway
Phagosome Formation
Agranulocyte Adhesion and Diapedesis
Granulocyte Adhesion and Diapedesis
Pathogen Induced Cytokine Storm Signaling Pathway
Role of Hypercytokinemia/hyperchemokinemia in the Pathogenesis of Influenza
Tumor Microenvironment Pathway
Atherosclerosis Signaling
Pathogen Induced Cytokine Storm Signaling Pathway
Agranulocyte Adhesion and Diapedesis
Granulocyte Adhesion and Diapedesis
Phagosome Formation
Airway Pathology in Chronic Obstructive Pulmonary Disease
S100 Family Signaling Pathway
Pathogen Induced Cytokine Storm Signaling Pathway
Agranulocyte Adhesion and Diapedesis
Granulocyte Adhesion and Diapedesis
IL-17 Signaling
Tumor Microenvironment Pathway
Pathogen Induced Cytokine Storm Signaling Pathway
Role of Chondrocytes in Rheumatoid Arthritis Signaling Pathway
Agranulocyte Adhesion and Diapedesis
Granulocyte Adhesion and Diapedesis
21 dpi
10 dpi
7-9 dpi
3-5 dpi
4.24
4.84
5.34
5.88
3.46
4.12
5.30
3.12
3.86
4.90
2.71
3.32
4
3.46
0
5
10
15
20
-log(p-value)
B
Differential Reg. of Cytokine Production in Intestinal Epithelial Cells by IL-17A...
Primary Immunodeficiency Signaling
S100 Family Signaling Pathway
Pathogen Induced Cytokine Storm Signaling Pathway
Agranulocyte Adhesion and Diapedesis
Granulocyte Adhesion and Diapedesis
Macrophage Classical Activation Signaling Pathway
Tumor Microenvironment Pathway
Agranulocyte Adhesion and Diapedesis
Role of Hypercytokinemia/hyperchemokinemia in the Pathogenesis of Influenza
Granulocyte Adhesion and Diapedesis
Pathogen Induced Cytokine Storm Signaling Pathway
Pathogenesis of Multiple Sclerosis
S100 Family Signaling Pathway
Airway Pathology in Chronic Obstructive Pulmonary Disease
Pathogen Induced Cytokine Storm Signaling Pathway
Agranulocyte Adhesion and Diapedesis
Granulocyte Adhesion and Diapedesis
Differential Reg. of Cytokine Production in Intestinal Epithelial Cells by IL-17A...
Pathogenesis of Multiple Sclerosis
Macrophage Classical Activation Signaling Pathway
Agranulocyte Adhesion and Diapedesis
Pathogen Induced Cytokine Storm Signaling Pathway
Granulocyte Adhesion and Diapedesis
21 dpi
10 dpi
7-9 dpi
3-5 dpi
2.83
4.42
4.95
2.67
3.46
3.55
2.12
3.27
2.45
4.32
2.32
0
5
10
15
20
-log(p-value)
